# Automated analysis of emotional expressions in dogs based on geometric morphometrics

**DOI:** 10.1038/s41598-025-15741-y

**Published:** 2025-09-02

**Authors:** George Martvel, Stefanie Riemer

**Affiliations:** 1 Tech4Animals Lab, University of Haifa, Abba Khoushy Ave 199, 3498838 Haifa, Israel; 2 Present Address: Messerli Research Institute, University of Veterinary Medicine, Veterinärplatz 1, 1210 Vienna, Austria

**Keywords:** Animal behaviour, Computer science

## Abstract

Automated analysis of facial expressions is a vibrant field in human affective computing, while research in nonhuman animals is still in its early stages. Compared to labour-intensive manual coding, automation can provide a more reliable and objective alternative, eliminating subjectivity and bias. However, using automated approaches of facial analysis in nonhuman animals “in the wild”, i.e. outside of controlled laboratory conditions, is a challenge given the nature of noisy datasets. Here we present the first study using a fully automated analysis of facial landmarks associated with different emotional states in a morphologically diverse sample of pet dogs. We applied a novel AI-pipeline to study fear expressions of dogs in their home environment, analysing owner-provided video recordings during a real-life firework situation on New Year’s Eve in comparison to a control evening without fireworks. Using a static geometric morphometrics-inspired analysis, the pipeline allows for quantifying dog facial expressions in an extremely noisy and diverse “in the wild” dataset, encompassing various breeds, angles and environments. We used an automated facial landmark system of 36 dog facial landmarks based on the Dog Facial Action Coding System. Due to the great variety in morphology of the included dogs, landmarks denoting the ear pinnae were excluded. Nonetheless, landmarks relating to the base of the ears differentiated most strongly between the conditions, suggesting backwards-drawn ears as the best indicator of the firework condition, which is in agreement with manually coded data. Additionally, the firework condition was associated with more mouth-opening, possibly reflecting panting in a subset of dogs. We conclude that automated analysis of dog facial expressions, based on the previously validated landmark system, is feasible in a diverse sample of pet dogs, paving the way towards automated emotion detection.

## Introduction

Emotions are considered to be central to the concept of animal welfare ^1^. They are affective responses elicited by specific stimuli of relevance to the individual. Such eliciting stimuli may be internal (e.g. the individual’s own behaviour or memory of past events) or external (e.g. the behaviour of other group members) ^2^. Emotions are commonly considered to encompass several components, including specific neural activation, physiological reactions, bodily expressions, a cognitive component (appraisal) and feelings ^3–8^. While subjective states (“feelings”) ^9,10^ could be considered at the core of animal welfare ^11–13^, they cannot be measured directly. Instead, proxy indicators, such as physiological changes (e.g. body temperature, heart rate, and hormone levels), behavioural reactions, vocalisations and bodily and facial expressions can be measured objectively, allowing inferences about the possible emotional experience of the animal ^14^.

To validate emotional indicators, it is essential to utilise target situations that very likely induce the emotion of interest ^15^. For instance, Gähwiler et al. ^16^ used a citizen science approach, collecting home videos of dogs during New Year’s Eve fireworks as compared to an evening without fireworks to study fear expressions in dogs, with noise fears constituting one of the most common behaviour problems in dogs, affecting up to half the pet dog population ^17–21^. Other studies presented selected stimuli in a controlled experimental environment to dogs to measure behavioural and expressive reactions related to fear ^22,23^. Using predefined ethograms, these studies identified fear-associated behaviours and expressions such as avoidance, a lowered body posture and tail, retreating, vocalisations, hiding, panting, and more subtle indicators such as backwards-directed ears and blinking ^22,23^.

Evidence is increasing that facial expressions of emotions are not limited to humans, but that affective states, motivations, intentions and communicative intent can be inferred from facial expressions also in nonhuman mammals ^24–27^. Unlike in humans, in non-human species, the positioning of the ears also plays a prominent role ^28–32^. Some studies have identified the neurobiological pathways associated with emotional expressions in animals, such as contraction of facial muscles and flattening of the ears when particular relevant stimuli are presented ^24,33^, further supporting the use of facial expressions as emotion indicators in animals.

While the universality of facial expressions of emotions is controversially debated in humans ^34–36^, some issues from human facial expression of emotion, e.g. the influence of social norms ^37^ or emotional deception ^5^ likely don’t apply to nonhuman animals. Moreover, research on primates shows that they have less control over their facial expressions compared to their motor behaviors ^38^. This suggests that facial expressions in nonhuman animals have the potential to serve as reliable indicators of their emotional states ^39^.

A relatively objective analysis of facial expressions was made possible by the development of facial action coding systems (FACS) ^40^, which are still considered standard in facial expression analysis. Originally developed for humans, FACS is anatomically based and systematic, describing movements of specific facial muscles ^41^. To date, FACS for several non-human species have been developed, including several primate species, horses, cats, and dogs ^42–47^.

Although the extensive anatomical and appearance diversity of domestic dogs ^48–51^ makes studying facial expression in this species particularly challenging, Dog Facial Action Coding System (DogFACS) is increasingly being used to identify emotional states in pet dogs ^15,28,52–54^, particularly in studies conducted in controlled environments where facial behavior can be more effectively captured and analyzed. These studies have demonstrated consistent associations of facial action units with emotional states such as positive anticipation, frustration and fear ^15,28,52–54^.

However, the reliance on manual labelling of expressions and behaviours represents a significant limitation. The coding process is labour-intensive, may require extensive human training and certification, and remains susceptible to a certain degree of human error or bias ^55^. Automation can provide a more reliable and objective alternative, eliminating subjectivity and bias, as well as allowing for work on a much larger scale. It is therefore not surprising that automated facial expression analysis is a vibrant field in human emotion research and affective computing, with numerous commercial software tools available, such as Noldus FaceReader ^56^ and Affectiva ^57^. In nonhuman animals, however, the automation of facial expression analysis is still understudied.

In the context of dogs, Boneh-Shitrit et al. ^58^ have made first steps towards automation of the detection of DogFACS action units in dogs of one breed (*Labrador Retriever*), utilizing ResNet50 ^59^ and Vision Transformer (ViT) ^60^ backbones over the facial images cropped from video frames, and concluding that more extensive and diverse datasets are crucial for adequately addressing this challenge. The issue with this approach is that it depends on visual characteristics and is thus hardly scalable. For example, a computer vision model that is trained on a particular appearance (range of skin or fur colours) may underperform when encountering different appearances, as has been shown in studies involving humans ^61,62^.

Algorithms that utilise facial landmarks present a promising alternative method for automated facial analysis. These algorithms are currently being developed for various species, including cats, dogs, horses, cattle, and sheep ^63–72^; however, many of these efforts have limitations. Typically, there is a limited amount of training data, a small number of landmarks, and a lack of justification for their placement concerning facial muscles, which is crucial for capturing the subtle facial changes necessary for affect recognition.

Although traditionally studied as separate fields, facial landmark detection shares a strong connection with geometric morphometric approaches (GMM) ^73^. This approach relies on the multivariate examination of landmark coordinates, thereby preserving their relative spatial configuration to depict shape accurately. Within GMM, shape differences are assessed primarily using Procrustes distance, calculated following the alignment of shapes through a Generalised Procrustes Analysis (GPA) ^74^. GMM is particularly adept at analysing shape variation across a range of biological structures, allowing for the detection of subtle morphological differences that are significant in developmental and evolutionary research ^75^.

Geometric morphometric-inspired approaches are gaining interest as tools for analysing facial changes as indicators of animal affective states, particularly in studies on pain in cats ^76,77^ and macaques ^78^. Since these methods rely on single-frame analysis, they are especially valuable when data collection occurs “in the wild”, making it challenging to capture a clear view of an animal’s face for an extended period. One obstacle in using GMM analysis for facial expression analysis, however, is the time-consuming manual annotation of facial landmarks ^65^. The second is the necessity to manually select “good” frames, where all facial features are clearly visible. If a computer vision model can be trained to detect a sufficient number of facial landmarks automatically, this will solve both of these challenges: it does not require manual efforts, and the detection is robust to noise and obstruction, as frames of low quality can be automatically detected and discarded.

Recently, Martvel et al. addressed this gap in cat facial analysis by introducing a dataset containing 2,091 cat images and a model that detects 48 anatomy-based cat facial landmarks ^79^. This detector has shown strong performance in tasks that require nuanced facial analysis, such as recognising breed, cephalic type, and pain ^80,81^. Furthermore, Martvel et al. extended this approach to dogs by developing a comprehensive landmark scheme that includes 46 facial landmarks based on DogFACS, and a dataset of 3,274 images of dogs of 120 breeds ^82^. Based on this dataset, automated landmark detection models can be trained to analyse new data.

In the current study, we performed automated analysis of dogs’ facial expressions, based on the landmark system by Martvel et al. ^82^, to study dogs’ facial expressions in a fear situation during a real-life firework event as compared to a control situation without fireworks. We used recordings of 11 dogs from the dataset collected by Gähwiler et al. ^16^ from dog owners using a citizen science approach. An ethogram-based analysis in the original paper revealed that a backwards-directed ear position, measured at the base of the ear rather than the ear pinna, was most closely associated with the fireworks condition. Additionally, the durations of locomotion and panting were significantly greater during the fireworks than in the control setting. While there were increases in vocalisations, blinking, and hiding during the fireworks displays, these changes were no longer statistically significant after correcting for multiple testing. The extremely noisy and diverse data (ranging over dog breeds, environments, and angles) presents an interesting challenge for studying automated analysis, as dynamic landmark-based approaches extracting a continuous signal (like those used in Martvel et al. ^80^) are not feasible. Instead, we developed an automated end-to-end AI pipeline for (1) extracting informative frames from the noisy data and (2) detecting facial landmarks on the dog faces in the extracted frames, using this information to compare dog faces in fireworks and control conditions.

## Methods

### Dataset

**Fig. 1:**
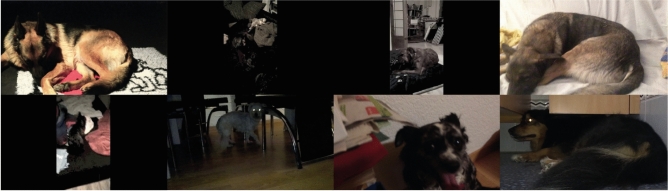
Random frames containing dogs from the original dataset of Gähwiler et al. ^16^ shared with permission of the owners.

The dataset for this study was collected previously by Gähwiler et al. ^16^ to investigate the behaviour of pet dogs during a real-life firework situation (approved by the Veterinary Office of the Canton of Bern, Switzerland, Licence number BE28/17). In a citizen science study, volunteer dog owners were asked to film their dog for five minutes (1) during fireworks on New Year’s Eve (firework video) and (2) again several days later at a similar time in the evening (control video) of what they considered their dog’s normal behaviour. All videos were taken indoors in the dogs’ homes. Although the authors did not specifically ask about the identity of the people present during the videos, no more than two people (including the operator) could be discerned in any of the submitted videos. The videos of 42 dogs (one video from each condition, fireworks and control) were initially included in the study, with an average length of $4.41 \pm 1.74$ minutes and 25-30 fps (see example frames in Fig. 1). More details about the data can be found in the original study of Gähwiler et al. ^16^

### Landmark detection

We processed all 84 videos (two videos for each of 42 dogs) using the Ensemble Landmark Detector (ELD) ^79^, trained on the DogFLW dataset ^82^. The output time series contained the frame’s timestamp, coordinates of 46 facial landmarks, and the model’s confidence (from 0 to 1), representing the quality of detected landmarks. The confidence was obtained from an output of the custom fully connected neural network classifier, trained on the DogFLW landmarks as positive examples and landmark predictions on random images not containing dog faces as negative examples. By giving a landmark set to this model, we obtained a classification score (softmax output) and interpreted it as a metric of “goodness” of the landmark set.

Depending on the video’s fps, the landmark sets were collected from every 2nd to 3rd frame, resulting in 12-15 landmark sets per second. All landmark sets were saved to csv files and then processed using the Python pandas library.

**Fig. 2:**
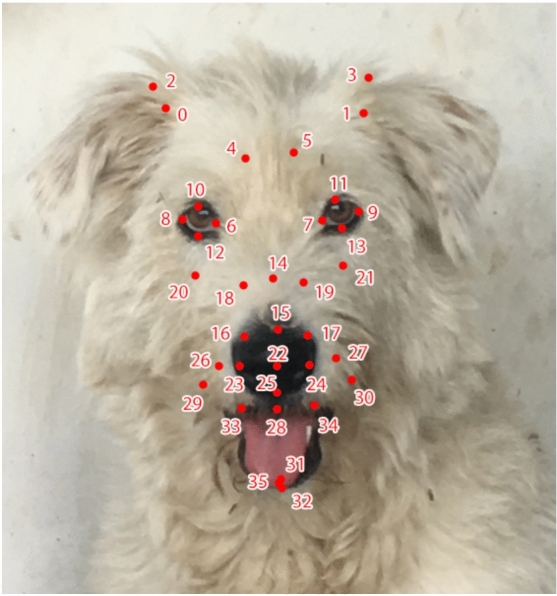
36 landmarks automatically detected on a dog’s face. Ear landmarks from the original scheme are discarded due to ear shape variation.

It is important to note that the subjects included various breeds with different ear shapes, ranging from upright pointy ears to cocked ears, rose ears, and floppy ears (see Supplementary Table 1). To mitigate the impact of this variance, we discarded five landmarks for each ear from the original scheme, leaving only ear base landmarks. This way, we ensured that ear movements were still considered in the landmark scheme and reactive ear movements (due to inertia, etc.) were mitigated. The final landmark scheme is shown in Fig. 2.

### Statistical analysis

To perform the statistical analysis, we followed the pipeline suggested by Finka et al. ^77^ and Gris et al. ^78^ First, we filtered landmark sets in all videos by confidence threshold and then by tilt and rotation angles, removing outliers by the 95th percentile. For the confidence threshold, a value of 0.6 was chosen based on the data quality-quantity tradeoff, leaving 15% of the original frames. Head tilt was measured as the angle between the line connecting the inner eye corners (landmarks 6 and 7) and the x-axis, and rotation was measured as the distance difference between the inner eye corners and the nose centre (landmark 22). Then, we filtered landmark sets within videos, applying an inter-sample interval of at least 1 second between sets to ensure the independence of dynamic expressions ^27^. All filtering steps were performed to exclude the poorly detected and repetitive landmark sets, while keeping the most informative ones.

After the initial filtering, we selected only individuals with at least ten landmark sets in each condition, resulting in 11 dogs out of the originally 42 dogs (see Supplementary Table 1 for details on breeds, age, and ear types). This reduction in sample size was due to the great variability in video quality, angles of recording, dogs hiding during the fireworks condition, or issues with a lack of contrast (e.g., a dark dog on a dark background), which made it difficult to detect facial features in many frames (see Fig. 1 for examples). Exclusions were fully automated and based on a confidence threshold for the landmark detection process. The top 10 landmark sets for individuals with more samples were selected based on the verification model’s confidence to ensure that we used the best images available, while maintaining an automated approach and keeping a dataset size appropriate for the statistical analysis.

Procrustes superimposition was applied to all the landmark coordinates to eliminate scaling, translation, and rotation effects between individuals and poses. Then, we performed a PCA on the Procrustes coordinates to produce a pattern of shape variation. Each principal component was assessed for its contribution to the overall variability, ensuring that we retained the most informative components for subsequent analyses.

To evaluate the impact of condition (firework/control) on the most informative principal components, we conducted an Analysis of Variance (ANOVA) on the PC scores, testing for statistically significant differences in their scores between the firework and control conditions. The normality of the residuals was examined using a Shapiro-Wilk test. Because the principal components were individually tested, we applied the Benjamini-Hochberg False Discovery Rate (FDR) correction to control explicitly for multiple comparisons. All tests were performed using the statsmodels Python library.

Given the reduction in sample size, we conducted a post-hoc power analysis to ensure sufficient statistical power for detecting meaningful differences. This analysis was performed at the individual-subject level, averaging PCA scores across multiple frames per dog to maintain statistical independence.

To remove the potential impact of the general head position, we examined the shape variance of the first three PCs, and applied the within-group pooled regression, since the first three PCs could be particularly associated with head-turning, nodding, or a combination of both ^78^. We then investigated the shape change between conditions using the residuals produced by the regression.

Discriminant analysis was performed to identify shape differences associated with experimental conditions. Canonical variates analysis (CVA) was applied, and the resulting scores were used to visualise the distribution of shapes along the discriminant axis. As part of this analysis, Wilks’ Lambda was calculated to assess the degree of separation between groups. Discriminant analysis was performed using scikit-learn’s LinearDiscriminantAnalysis.

## Results

**Fig. 3:**
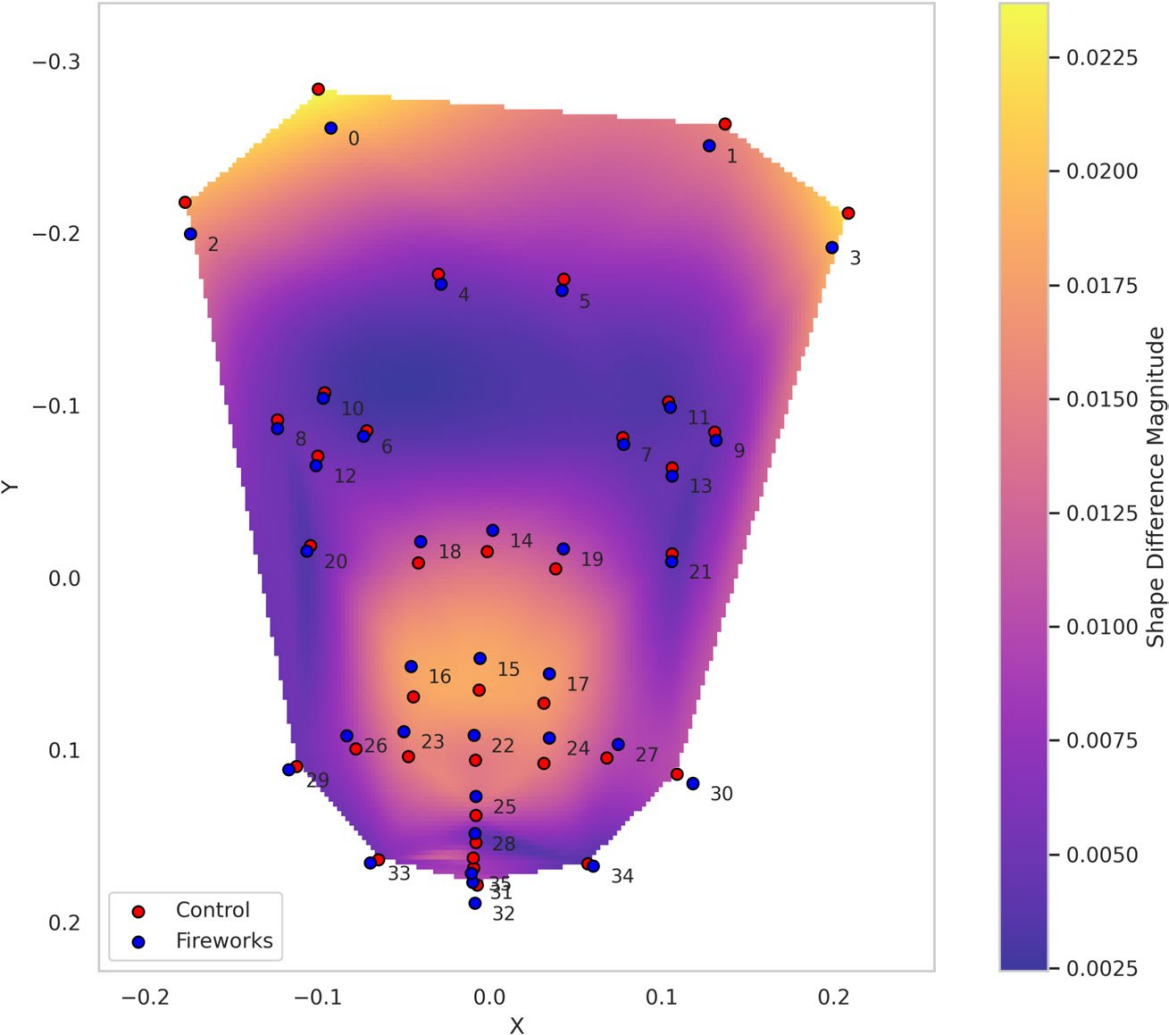
Mean position of 36 facial landmarks in fireworks and control conditions for all dogs and a heatmap of shape difference values.

220 landmark sets across two conditions were extracted from the eleven dog subjects in two conditions (10 per dog per condition). Based on the aligned landmarks (after the Procrustes superimposition), mean positions in each condition were calculated, and the shape difference was interpolated (see Fig. 3).

**Fig. 4:**
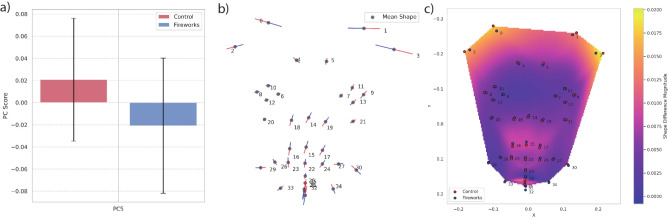
**a)** Mean PC5 values with standard deviation for dogs in fireworks and control conditions; **b)** Mean face shape with relative changes of PC5. The red colour represents the increase by 1 SD from the mean value, and the blue colour represents the decrease by 1 SD; **c)** Mean position of 36 facial landmarks in fireworks and control conditions, and a heatmap of shape difference values after removing variation of head orientation.

The first eight principal components explained 90% of variance within the population (see Supplementary Figure 1 for cumulative explained variance), with individual contributions ranging from 41% (PC1) to 2% (PC8) and subsequent components each contributing less than 2%. Across the eight PC components tested, only PC5 ($F=13.57, p=0.004$) showed significant differences between the conditions (see Supplementary Table 2 for details). After correction, PC5 remained statistically significant (corrected $p = 0.034$), confirming that differences observed in this component were robust. Figure 4a) shows mean PC5 values in fireworks and control conditions, while Fig. 4b) demonstrates the change of mean face shape with the change of PC 5, which correlates with Fig. 3.

To validate the statistical power, we calculated the mean differences between the fireworks and control conditions per subject for PC5. The observed effect size was large (paired Cohen’s $d = 1.11$), resulting in a statistical power of 91.2%. To confirm the normality of residuals, we conducted a Shapiro-Wilk test, which showed no significant deviations from normality for PC5 ($W = 0.957$, $p = 0.434$).

By calculating the residuals of the within-group pooled regression between Procrustes coordinates and the first three PCs, we mitigated the head rotation impact on the mean shapes, as suggested by Gris et al. ^78^. The resulting PC variations and mean shapes indicated the ears were moved medially during the fireworks condition relative to the control condition, which could be interpreted as drawn-back ears (see Fig. 4c). Only slight differences in brows and eye shape were apparent, although the brows seem slightly lowered during fireworks, and the eyes appear to be oriented slightly more medially during fireworks. Meanwhile, there are some noticeable differences in the landmarks designating muzzle shape, with the upper landmarks (nose landmarks, as well as landmarks 25 and 28) in a more dorsal position and the lower landmarks (31 and 32) in a more ventral position during fireworks compared to the control situation. This would be consistent with mouth-opening, such as in panting or yawning. Furthermore, the edges of the lips (landmarks 33 and 34) are more lateral during fireworks, which could indicate panting.

**Fig. 5:**
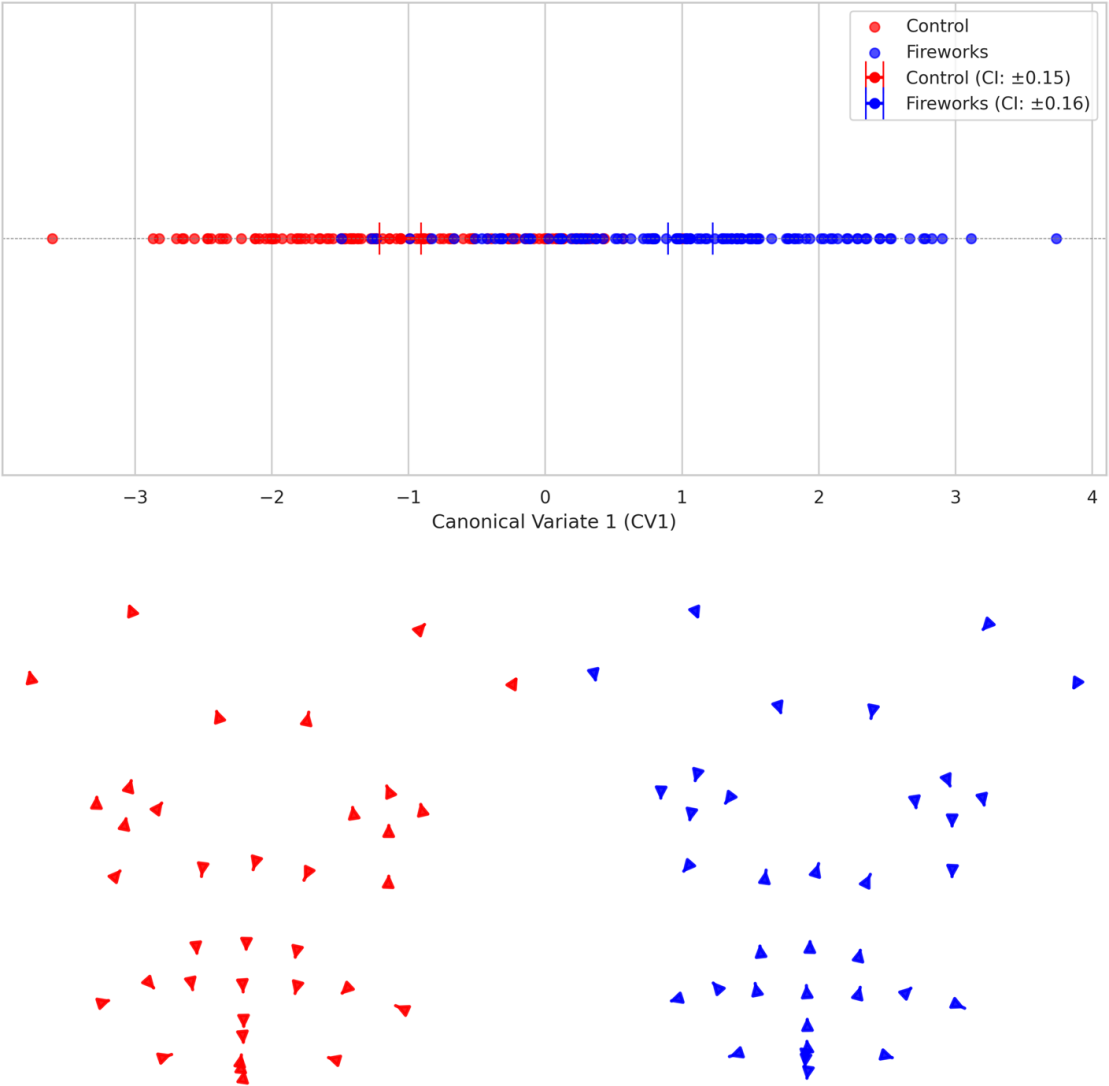
Facial shape variation between conditions after Canonical Variate Analysis. Each colour refers to one condition and 0.9 confidence intervals (CI). The scatterplots below depict changes in shape along the canonical variate CV1 (arrows show the directions from the general mean to the mean of each condition: control on the left (red) and fireworks on the right (blue)).

Discriminant analysis was also used to locate differences related to the condition. A CVA scatterplot (Figure 5 (top)) shows differences in facial shape between conditions. The overall multivariate Wilks’ Lambda for group separation was 0.058 (approximate *F* = 41.43, $df_1$ = 72, $df_2$ = 183, $p < 0.0001$), indicating a highly significant difference in facial shape between conditions. Moreover, the changes in shape along CV1 demonstrate differences similar to those in the PCA (Fig. 5 (bottom)).

## Discussion

**Fig. 6:**
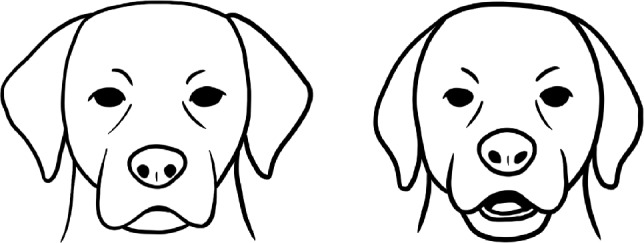
Schematic representation of the dogs’ facial expressions: neutral (left), and stressed (right), according to the morphometric analysis.

This paper is the first to apply a fully automated geometric morphometrics-inspired analysis to study dog facial expressions in a fear context. This approach allowed us to deal with the challenging and noisy nature of data obtained outside of standardised laboratory conditions (in people’s homes during fireworks vs an evening without fireworks). Indeed, only around 40% of videos fulfilled the requirement of dog faces detected in more than 50% of frames, and only one video had a dog’s face on each frame. The AI-based approach allowed us to effectively and automatically filter informative frames containing actual facial expressions for the analysis. In practice, this led to a reduction in sample size from 42 dogs in the initial dataset to only 11 dogs. Despite the relatively low amount of extracted informative frames, we discovered consistent differences in dogs’ facial shapes between the conditions. This demonstrates the value of the proposed methodology for other noisy “in-the-wild” datasets for studying animal facial expressions in different emotional states and other contexts. Figure 6 shows the schematic representation of stress-related facial expressions identified in this study.

Notably, due to the great variety in morphology of the dogs included, we decided to remove the landmarks representing the ear pinnae from the analysis. This led to certain information loss, and therefore it was not possible for us to analyse differences in ear positions directly (such as in Finka et al. ^77^). Nonetheless, using landmarks on the ear bases indicated that ears were the features that differed most strongly between the fireworks and the control condition: during fireworks, the ears were held more medially, which can be interpreted as being drawn backwards. This result is consistent with the findings of Gähwiler et al ^16^. Like in the current study, they visually assessed ear position measured at the base of the ear to avoid inconsistencies due to the ear pinnae, and this measure differentiated most strongly of all included measures between the fireworks and the control condition. Our results thus indicate that ear position is a highly relevant potential stress indicator (see also Bremhorst et al. ^15,28^ on frustration) which can be reliably assessed by automated analysis in a diverse sample of domestic dogs.

Additionally, we found significant differences in landmarks associated with mouth-opening between the two conditions. Interestingly, using DogFACS coding to assess facial expressions of frustration vs positive anticipation, Bremhorst et al. found an association of frustration with the DogFACS action units “lips part” and “jaw drop” ^15^. Additionally, they also recorded a higher frequency of the “lip corner puller” and “tongue show” during a frustration condition as compared with a positive anticipation condition. In the current study, we did not differentiate between possible different action units, although our results could possibly be interpreted in line with the findings of Bremhorst et al. ^15,28^ for frustration. Additionally, such a result could be potentially affected by barking, during which the mouth landmarks behave similarly.

Although the difference between landmarks indicating the aperture of the mouth is not extensive (Figure 3), it is possible that this finding reflects the incidence of panting in a small subset of dogs, as noted by Gähwiler et al. ^16^ Indeed, Caeiro et al. ^52^ discovered that dogs consistently showed a higher rate of AD126 (action descriptor, panting) during fear contexts. While the extent of mouth opening that can be inferred from the automated analysis in the current study might appear more subtle than panting, this could potentially be an artefact of the high inter-individual variability in reactions to the fireworks. As Gähwiler et al. ^16^ pointed out, only a small number of dogs showed panting, but with few exceptions, it occurred exclusively in the fireworks condition. Due to our averaging over all participating dogs, the extent of mouth-opening might appear smaller than it actually was in those few dogs that did pant.

This highlights the necessity of careful human interpretation when analysing AI-generated findings. Moreover, unlike panting, the wide opening of the mouth in Caeiro et al. ^52^ was significantly associated with states they interpreted as happiness. Thus, attention to detail and to inter-individual variability is paramount to consider in order to obtain valid interpretations from dogs’ facial expressions. Indeed, Gähwiler et al. ^16^ discussed the high variability in fear behaviour between individual dogs, stating that “individual differences must be taken into account when aiming to assess an individual’s level of fear, as relevant measures may not be the same for all individuals”. Universally, however, the fireworks condition was associated with backwards-directed ears, which represented the best indicator both according to manual coding and the current automated analysis.

As discussed in Bremhorst et al. ^15^, it is questionable to what extent we are currently able to differentiate negative emotions (such as fear and frustration) from facial expressions alone, or whether we can only draw conclusions about more universal stress expressions. For instance, studies indicate that backwards-directed ears, mouth-opening/panting and blinking are associated with both fear and frustration ^28,83–86^. Only Caeiro et al. ^52^ differentiated expressions associate with fear and frustration based on DogFACS; however, they only found very few (1–2) action units to be associated with the respective emotion states, possibly because their videos were obtained from social media showing dogs in diverse situations which the authors retrospectively categorised as inducing either fear or frustration. In contrast, the context was clearly defined in Bremhorst et al. ^15,28^ (positive anticipation vs frustration when a reward is delivered but inaccessible) and Gähwiler et al. ^16^ (presence or absence of fireworks), and within-subjects analyses were possible.

The only other behaviour that could be considered as a facial expression differentiating between the conditions reported in Gähwiler et al. was an increased frequency of blinking during the firework condition. Using the approach from the current study, this could not be confirmed, as the analysis was based on automatically selected snapshots of dogs’ faces, rather than continuous monitoring. Brief events, such as blinking or barking, could be missed using this approach.

An additional limitation of the static geometric morphometrics-inspired analysis used here is the usage of two-dimensional facial landmarks, since shape variations could potentially be caused not by conditions but by head rotation and perspective distortion. In the current study, we partially mitigated this by regressing Procrustes coordinates onto PCs and using residuals to remove head rotation effects, but the more advanced techniques with depth evaluation and adding the third dimension to landmarks may be needed to totally reduce the drawbacks of the current representation ^87,88^.

As Bremhorst et al. ^15^ pointed out, individual emotion indicators are generally not sufficient to predict emotions with a high degree of accuracy. However, machine learning offers an advantage by analysing multiple indicators simultaneously. In the current study, we highlighted only one way to “decipher” animal visual signals, based on the static approach. Reliability and accuracy of emotion assessments could be improved even further by taking into account behavioural information (such as freezing), bodily signals and posture, vocalisations, and possibly physiological measures such as heart rate, while also considering possible inter-individual variability in emotional expression.

To conclude, this pioneering work demonstrates that automated emotion assessment in morphologically diverse dogs “in the wild” is possible. Follow-up studies should extend this work and assess the accuracy of automated analyses for differentiating between situations of different valence (and thus animals’ likely emotional states) based on their facial expressions. Future potential applications of automated dog facial expression analysis include their use in clinical and behavioural studies, as well as implementation of such algorithms into the surveillance systems in veterinary clinics and shelters, where they can be used to detect and alert to certain animal behaviours and conditions based on visual cues in a fully automated manner.

## Supplementary Information

Supplementary Information.

## Data Availability

The original video dataset for this study was collected by Gähwiler et al. ^16^. The code generated during this study is available at https://github.com/martvelge/Fireworks_GMM. The landmark dataset generated and analysed during the current study is available from the corresponding author upon request.

## References

[1] Špinka, M. Social dimension of emotions and its implication for animal welfare. *Appl. Anim. Behav. Sci.* **138**, 170–181 (2012).

[2] Scherer, K. R. What are emotions? And how can they be measured?. *Soc. Sci. Inf.* **44**, 695–729 (2005).

[3] Scherer, K. R. On the nature and function of emotion: A component process approach. In *Approaches to Emotion*. 293–317 (Psychology Press, 2014).

[4] Paul, E. S., Sher, S., Tamietto, M., Winkielman, P. & Mendl, M. T. Towards a comparative science of emotion: Affect and consciousness in humans and animals. *Neurosci. Biobehav. Rev.* **108**, 749–770 (2020). 31778680 10.1016/j.neubiorev.2019.11.014 PMC6966324

[5] Anderson, D. J. & Adolphs, R. A framework for studying emotions across species. *Cell* **157**, 187–200 (2014). 24679535 10.1016/j.cell.2014.03.003 PMC4098837

[6] Baciadonna, L., Duepjan, S., Briefer, E. F., Padilla de la Torre, M. & Nawroth, C. Looking on the bright side of livestock emotions—The potential of their transmission to promote positive welfare. *Front. Vet. Sci.* **5**, 218 (2018). 30258847 10.3389/fvets.2018.00218 PMC6143710

[7] Tracy, J. L. & Randles, D. Four models of basic emotions: A review of Ekman and Cordaro, Izard, Levenson, and Panksepp and Watt. *Emot. Rev.* **3**, 397–405 (2011).

[8] Boissy, A. & Erhard, H. W. How studying interactions between animal emotions, cognition, and personality can contribute to improve farm animal welfare. In *Genetics and the Behavior of Domestic Animals*. 95–129 (Elsevier, 2014).

[9] Moors, A., Ellsworth, P. C., Scherer, K. R. & Frijda, N. H. Appraisal theories of emotion: State of the art and future development. *Emot. Rev.* **5**, 119–124 (2013).

[10] Panksepp, J. The basic emotional circuits of mammalian brains: Do animals have affective lives?. *Neurosci. Biobehav. Rev.* **35**, 1791–1804 (2011). 21872619 10.1016/j.neubiorev.2011.08.003

[11] Duncan, I. J. A concept of welfare based on feelings. *The Well-Being of Farm Animals: Challenges and Solutions*. 85–101 (2004).

[12] Fraser, D. Understanding animal welfare. *Acta Vet. Scand.* **50**, S1 (2008). 19049678 10.1186/1751-0147-50-S1-S1 PMC4235121

[13] Dawkins, M. S. The science of animal suffering. *Ethology* **114**, 937–945 (2008).

[14] Mendl, M., Burman, O. H. & Paul, E. S. An integrative and functional framework for the study of animal emotion and mood. *Proc. R. Soc. B Biol. Sci.* **277**, 2895–2904 (2010). 10.1098/rspb.2010.0303 PMC2982018 20685706

[15] Bremhorst, A., Mills, D., Würbel, H. & Riemer, S. Evaluating the accuracy of facial expressions as emotion indicators across contexts in dogs. *Anim. Cognit.* 1–16 (2021). 10.1007/s10071-021-01532-1 PMC8904359 34338869

[16] Gähwiler, S., Bremhorst, A., Tóth, K. & Riemer, S. Fear expressions of dogs during new year fireworks: A video analysis. *Sci. Rep.* **10**, 16035 (2020). 32994423 10.1038/s41598-020-72841-7 PMC7525486

[17] Riemer, S. Not a one-way road—Severity, progression and prevention of firework fears in dogs. *PLoS One* **14**, e0218150 (2019). 31490926 10.1371/journal.pone.0218150 PMC6730926

[18] Riemer, S. Effectiveness of treatments for firework fears in dogs. *J. Vet. Behav.* **37**, 61–70 (2020).

[19] Riemer, S. Therapy and prevention of noise fears in dogs—A review of the current evidence for practitioners. *Animals* **13**, 3664 (2023). 38067015 10.3390/ani13233664 PMC10705068

[20] Blackwell, E. J., Bradshaw, J. W. & Casey, R. A. Fear responses to noises in domestic dogs: Prevalence, risk factors and co-occurrence with other fear related behaviour. *Appl. Anim. Behav. Sci.* **145**, 15–25 (2013).

[21] Dale, A., Walker, J., Farnworth, M., Morrissey, S. & Waran, N. A survey of owners’ perceptions of fear of fireworks in a sample of dogs and cats in New Zealand. *N. Z. Vet. J.* **58**, 286–291 (2010). 21151214 10.1080/00480169.2010.69403

[22] Stellato, A. C., Flint, H. E., Widowski, T. M., Serpell, J. A. & Niel, L. Assessment of fear-related behaviours displayed by companion dogs (Canis familiaris) in response to social and non-social stimuli. *Appl. Anim. Behav. Sci.* **188**, 84–90 (2017).

[23] Flint, H. E., Coe, J. B., Serpell, J. A., Pearl, D. L. & Niel, L. Identification of fear behaviors shown by puppies in response to nonsocial stimuli. *J. Vet. Behav.* **28**, 17–24 (2018).

[24] Mota-Rojas, D. et al. How facial expressions reveal acute pain in domestic animals with facial pain scales as a diagnostic tool. *Front. Vet. Sci.* **12**, 1546719 (2025). 40104548 10.3389/fvets.2025.1546719 PMC11913824

[25] Dalla Costa, E. et al. Development of the horse grimace scale (HGS) as a pain assessment tool in horses undergoing routine castration. *PLOS ONE* **9**, e92281 (2014). 24647606 10.1371/journal.pone.0092281 PMC3960217

[26] Wathan, J., Burrows, A. M., Waller, B. M. & McComb, K. Equifacs: The equine facial action coding system. *PLOS ONE* **10**, e0131738 (2015). 26244573 10.1371/journal.pone.0131738 PMC4526551

[27] Bennett, V., Gourkow, N. & Mills, D. S. Facial correlates of emotional behaviour in the domestic cat (Felis catus). *Behav. Process.* **141**, 342–350 (2017). 10.1016/j.beproc.2017.03.011 28341145

[28] Bremhorst, A., Sutter, N. A., Würbel, H., Mills, D. S. & Riemer, S. Differences in facial expressions during positive anticipation and frustration in dogs awaiting a reward. *Sci. Rep.* **9**, 1–13 (2019). 31848389 10.1038/s41598-019-55714-6 PMC6917793

[29] Boissy, A. et al. Cognitive sciences to relate ear postures to emotions in sheep. *Anim. Welfare* **20**, 47–56 (2011).

[30] Battini, M., Agostini, A. & Mattiello, S. Understanding cows’ emotions on farm: Are eye white and ear posture reliable indicators?. *Animals* **9**, 477 (2019). 31344842 10.3390/ani9080477 PMC6720764

[31] Lambert, H. & Carder, G. Positive and negative emotions in dairy cows: Can ear postures be used as a measure?. *Behav. Process.* **158**, 172–180 (2019). 10.1016/j.beproc.2018.12.007 30543843

[32] Bremhorst, A., Mills, D., Würbel, H. & Riemer, S. Evaluating the accuracy of facial expressions as emotion indicators across contexts in dogs. *Anim. Cognit.* **25**, 121–136 (2022). 34338869 10.1007/s10071-021-01532-1 PMC8904359

[33] Dolensek, N., Gehrlach, D. A., Klein, A. S. & Gogolla, N. Facial expressions of emotion states and their neuronal correlates in mice. *Science* **368**, 89–94 (2020). 32241948 10.1126/science.aaz9468

[34] Ekman, P. & Keltner, D. Universal facial expressions of emotion. In * Nonverbal Communication: Where Nature Meets Culture* (Segerstrale, U. & Molnar, P. eds.) . Vol. 27. 46 (1997).

[35] Russell, J. A., Bachorowski, J.-A. & Fernández-Dols, J.-M. Facial and vocal expressions of emotion. *Annu. Rev. Psychol.* **54**, 329–349 (2003). 12415074 10.1146/annurev.psych.54.101601.145102

[36] Wolf, K. Measuring facial expression of emotion. *Dial. Clin. Neurosci.* **17**, 457–462 (2015). 10.31887/DCNS.2015.17.4/kwolf PMC4734883 26869846

[37] Hess, U. & Thibault, P. Darwin and emotion expression. *Am. Psychol.* **64**, 120 (2009). 19203144 10.1037/a0013386

[38] Descovich, K. A. et al. Facial expression: An under-utilised tool for the assessment of welfare in mammals. *Altex* (2017). 10.14573/altex.1607161 28214916

[39] Mota-Rojas, D. et al. Current advances in assessment of dog’s emotions, facial expressions, and their use for clinical recognition of pain. *Animals* **11**, 3334 (2021). 34828066 10.3390/ani11113334 PMC8614696

[40] Ekman, P. & Friesen, W. Facial action coding system: A technique for the measurement of facial movement. *Environ. Psychol. Nonverb. Behav.* (1978).

[41] Waller, B., Julle-Daniere, E. & Micheletta, J. Measuring the evolution of facial ‘expression’ using multi-species facs. *Neurosci. Biobehav. Rev.* **113**, 1–11 (2020). 32105704 10.1016/j.neubiorev.2020.02.031

[42] Caeiro, C., Waller, B., Zimmerman, E., Burrows, A. & Davila Ross, M. Orangfacs: A muscle-based movement coding system for facial communication in orangutans. *Int. J. Primatol.* **34**, 115–129 (2013).

[43] Parr, L. A., Waller, B. M., Vick, S. J. & Bard, K. A. Classifying chimpanzee facial expressions using muscle action. *Emotion* **7**, 172 (2007). 17352572 10.1037/1528-3542.7.1.172 PMC2826116

[44] Clark, P. R. et al. Morphological variants of silent bared-teeth displays have different social interaction outcomes in crested macaques (Macaca nigra). *Am. J. Phys. Anthropol.* **173**, 411–422 (2020). 32820559 10.1002/ajpa.24129

[45] Correia-Caeiro, C., Holmes, K. & Miyabe-Nishiwaki, T. Extending the MaqFACS to measure facial movement in Japanese macaques (Macaca fuscata) reveals a wide repertoire potential. *PLOS ONE* **16**, e0245117 (2021). 33411716 10.1371/journal.pone.0245117 PMC7790396

[46] Waller, B. et al. *DogFACS: The Dog Facial Action Coding System* (University of Portsmouth, 2013).

[47] Caeiro, C. C., Burrows, A. M. & Waller, B. M. Development and application of catfacs: Are human cat adopters influenced by cat facial expressions? *Appl. Anim. Behav. Sci.* (2017).

[48] Georgevsky, D., Carrasco, J. J., Valenzuela, M. & McGreevy, P. D. Domestic dog skull diversity across breeds, breed groupings, and genetic clusters. *J. Vet. Behav.* **9**, 228–234 (2014).

[49] Drake, A. G. & Klingenberg, C. P. Large-scale diversification of skull shape in domestic dogs: Disparity and modularity. *Am. Nat.* **175**, 289–301 (2010). 20095825 10.1086/650372

[50] Burrows, A. M. et al. Dog faces exhibit anatomical differences in comparison to other domestic animals. *Anat. Rec.* **304**, 231–241 (2021). 10.1002/ar.24507 32969196

[51] Hobkirk, E. R. & Twiss, S. D. Domestication constrains the ability of dogs to convey emotions via facial expressions in comparison to their wolf ancestors. *Sci. Rep.* **14**, 10491 (2024). 38714729 10.1038/s41598-024-61110-6 PMC11076640

[52] Caeiro, C., Guo, K. & Mills, D. Dogs and humans respond to emotionally competent stimuli by producing different facial actions. *Sci. Rep.* **7**, 1–11 (2017). 29138393 10.1038/s41598-017-15091-4 PMC5686192

[53] Pedretti, G. et al. Audience effect on domestic dogs’ behavioural displays and facial expressions. *Sci. Rep.* **12**, 1–13 (2022). 35697913 10.1038/s41598-022-13566-7 PMC9192729

[54] Sexton, C. L. et al. What is written on a dog’s face? Evaluating the impact of facial phenotypes on communication between humans and canines. *Animals* **13**, 2385 (2023). 37508162 10.3390/ani13142385 PMC10376741

[55] Hamm, J., Kohler, C. G., Gur, R. C. & Verma, R. Automated facial action coding system for dynamic analysis of facial expressions in neuropsychiatric disorders. *J. Neurosci. Methods* **200**, 237–256 (2011). 21741407 10.1016/j.jneumeth.2011.06.023 PMC3402717

[56] den Uyl, M. J. & van Kuilenburg, H. The facereader: Online facial expression recognition. In *Proceedings of Measuring Behavior*. 589–590 (2005).

[57] iMotions. Affectiva imotions biometric research platform. https://imotions.com/emotient/ (2015). Accessed 15 Apr 2025.

[58] Boneh-Shitrit, T. et al. Explainable automated recognition of emotional states from canine facial expressions: The case of positive anticipation and frustration. *Sci. Rep.* **12**, 22611 (2022). 36585439 10.1038/s41598-022-27079-w PMC9803655

[59] He, K., Zhang, X., Ren, S. & Sun, J. Deep residual learning for image recognition. In *CVPR* (2016).

[60] Dosovitskiy, A. et al. An image is worth 16x16 words: Transformers for image recognition at scale. In *ICLR* (2021).

[61] Birhane, A. The Unseen Black Faces of AI Algorithms (2022). 10.1038/d41586-022-03050-7 36261566

[62] Klare, B. F., Burge, M. J., Klontz, J. C., Bruegge, R. W. V. & Jain, A. K. Face recognition performance: Role of demographic information. *IEEE Trans. Inf. For. Secur.* **7**, 1789–1801 (2012).

[63] Pessanha, F., Salah, A. A., van Loon, T. & Veltkamp, R. Facial image-based automatic assessment of equine pain. *IEEE Trans. Affect. Comput.*10.1109/TAFFC.2022.3177639 (2022).

[64] Liu, J., Kanazawa, A., Jacobs, D. & Belhumeur, P. Dog breed classification using part localization. In *Computer Vision–ECCV 2012: 12th European Conference on Computer Vision, Florence, Italy, October 7–13, 2012, Proceedings, Part I 12*. 172–185 (Springer, 2012).

[65] Khan, M. H. et al. Animalweb: A large-scale hierarchical dataset of annotated animal faces. In *Proceedings of the IEEE/CVF Conference on Computer Vision and Pattern Recognition*. 6939–6948 (2020).

[66] Zhang, W., Sun, J. & Tang, X. Cat head detection-how to effectively exploit shape and texture features. In *Computer Vision–ECCV 2008: 10th European Conference on Computer Vision, Marseille, France, October 12-18, 2008, Proceedings, Part IV 10*. 802–816 (Springer, 2008).

[67] Cao, J. et al. Cross-domain adaptation for animal pose estimation. In *Proceedings of the IEEE/CVF International Conference on Computer Vision*. 9498–9507 (2019).

[68] Coffman, E. et al. Cattleface-rgbt: Rgb-t cattle facial landmark benchmark. arXiv preprint arXiv:2406.03431 (2024).

[69] Mougeot, G., Li, D. & Jia, S. A deep learning approach for dog face verification and recognition. In *PRICAI 2019: Trends in Artificial Intelligence: 16th Pacific Rim International Conference on Artificial Intelligence, Cuvu, Yanuca Island, Fiji, August 26-30, 2019, Proceedings, Part III 16*. 418–430 (Springer, 2019).

[70] Sun, Y. & Murata, N. Cafm: A 3D morphable model for animals. In *Proceedings of the IEEE/CVF Winter Conference on Applications of Computer Vision Workshops*. 20–24 (2020).

[71] Hewitt, C. & Mahmoud, M. Pose-informed face alignment for extreme head pose variations in animals. In *2019 8th International Conference on Affective Computing and Intelligent Interaction (ACII)*. 1–6 (IEEE, 2019).

[72] Yang, H., Zhang, R. & Robinson, P. Human and sheep facial landmarks localisation by triplet interpolated features. In *2016 IEEE Winter Conference on Applications of Computer Vision (WACV)*. 1–8 (IEEE, 2016).

[73] Mitteroecker, P. & Gunz, P. Advances in geometric morphometrics. *Evolut. Biol.* **36**, 235–247. 10.1007/s11692-009-9055-x (2009).

[74] Gower, J. C. Generalized procrustes analysis. *Psychometrika* **40**, 33–51. 10.1007/BF02291478 (1975).

[75] Mitteröcker, P. Morphometrics in evolutionary developmental biology. In *Evolutionary Developmental Biology: A Reference Guide* (Nuño de la Rosa, L. & Müller, G. B. eds.). 941–951. 10.1007/978-3-319-33038-9_119 (Springer, 2021).

[76] Holden, E. et al. Evaluation of facial expression in acute pain in cats. *J. Small Anim. Pract.* **55**, 615–621 (2014). 25354833 10.1111/jsap.12283

[77] Finka, L. R. et al. Geometric morphometrics for the study of facial expressions in non-human animals, using the domestic cat as an exemplar. *Sci. Rep.* **9**, 1–12 (2019). 31285531 10.1038/s41598-019-46330-5 PMC6614427

[78] Gris, V. N. et al. Author correction: Investigating subtle changes in facial expression to assess acute pain in Japanese macaques. *Sci. Rep.* **13**, 12931. 10.1038/s41598-023-40053-4 (2023). 37558672 10.1038/s41598-023-40053-4 PMC10412631

[79] Martvel, G., Shimshoni, I. & Zamansky, A. Automated detection of cat facial landmarks. *Int. J. Comput. Vis.* **132**, 3103–3118. 10.1007/s11263-024-02006-w (2024).

[80] Martvel, G. et al. Automated video-based pain recognition in cats using facial landmarks. *Sci. Rep.* **14**, 28006. 10.1038/s41598-024-78406-2 (2024). 39543343 10.1038/s41598-024-78406-2 PMC11564822

[81] Martvel, G. et al. Automated landmark-based cat facial analysis and its applications. *Front. Vet. Sci.* **11**, 1442634. 10.3389/fvets.2024.1442634 (2024). 39717789 10.3389/fvets.2024.1442634 PMC11663861

[82] Martvel, G. et al. Dogflw: Dog facial landmarks in the wild dataset. arXiv preprint arXiv:2405.11501 (2024).

[83] Gruen, M. E. et al. The use of an open-field model to assess sound-induced fear and anxiety-associated behaviors in labrador retrievers. *J. Vet. Behav.* **10**, 338–345. 10.1016/j.jveb.2015.03.007 (2015). 26273235 10.1016/j.jveb.2015.03.007 PMC4530634

[84] Sheppard, G. & Mills, D. S. Evaluation of dog-appeasing pheromone as a potential treatment for dogs fearful of fireworks. *Vet. Rec.* **152**, 432–436. 10.1136/vr.152.14.432 (2003). 12708592 10.1136/vr.152.14.432

[85] Dreschel, N. A. & Granger, D. A. Physiological and behavioral reactivity to stress in thunderstorm-phobic dogs and their caregivers. *Appl. Anim. Behav. Sci.* **95**, 153–168. 10.1016/j.applanim.2005.04.009 (2005).

[86] Franzini de Souza, C. C., Maccariello, C. E. M., Dias, D. P. M., dos Santos Almeida, N. A. & de Medeiros, M. A. Autonomic, endocrine and behavioural responses to thunder in laboratory and companion dogs. *Physiol. Behav.* **169**, 208–215. 10.1016/j.physbeh.2016.12.006 (2017). 27939362 10.1016/j.physbeh.2016.12.006

[87] Jeni, L. A., Cohn, J. F. & Kanade, T. Dense 3d face alignment from 2d videos in real-time. In *2015 11th IEEE International Conference and Workshops on Automatic Face and Gesture Recognition (FG)*. Vol. 1. 1–8. 10.1109/FG.2015.7163081 (IEEE, 2015). 10.1109/FG.2015.7163142 PMC4900890 27293385

[88] Yu, Y., Funes Mora, K. A. & Odobez, J.-M. Robust and accurate 3d head pose estimation through 3dmm and online head model reconstruction. In *2017 12th IEEE International Conference on Automatic Face and Gesture Recognition (FG 2017)*. 711–718. 10.1109/FG.2017.86 (IEEE, 2017).

