# Supplementary material for: Automated analysis of emotional expressions in dogs based on geometric morphometrics
Source: Sci Rep. 2025 Sep 2;15:32331. doi: 10.1038/s41598-025-15741-y (PMC12405473; doi:10.1038/s41598-025-15741-y)
Supplement: Supplementary file 1 — Supplementary Information. [file 41598_2025_15741_MOESM1_ESM.docx]

**Automated Analysis of Emotional Expressions in Dogs based on AI-Enhanced Geometric Morphometrics**

**George Martvel^1,@^ and Stefanie Riemer^2^**

^1^ Tech4Animals Lab, University of Haifa, Abba Khoushy Ave 199, 3498838 Haifa, Israel

^2^ Messerli Research Institute, University of Veterinary Medicine, Vienna, Veterinärplatz 1, 1210 Vienna, Austria

^@^ [martvelge@gmail.com](mailto:martvelge@gmail.com)

**Supplementary Information**

**Supplementary Table 1.** Demographic information about the subjects, whether they were included in the analysis (sufficient frames with facial landmarks detected), and the ear shape of included dogs.

| **Breed** | **Age (years)** | **Sex** | **Neuter status** | **Included in analysis** | **Ear shape** |
| --- | --- | --- | --- | --- | --- |
| Mixed breed | 3.92 | Female | Neutered | Yes | rose ears |
| Mixed breed | 6.42 | Male | Neutered | Yes | rose ears |
| Shiba Inu | 3.46 | Female | Intact | Yes | pointy, upright |
| Mixed breed | 4.34 | Male | Neutered | Yes | floppy |
| Mixed breed | 9.33 | Male | Neutered | Yes | rose ears |
| Chihuahua | 6.63 | Male | Neutered | Yes | pointy, upright |
| Australian Shepherd | 3.67 | Male | Chemical castration | Yes | cocked |
| German Shepherd | 7.45 | Male | Neutered | Yes | pointy, upright |
| Coton de Tuléar | 8.88 | Female | Neutered | Yes | button ears |
| Chihuahua | 4.81 | Female | Neutered | Yes | pointy, upright |
| Mixed breed | 2.67 | Female | Neutered | Yes | floppy |
| Border Collie | 6.32 | Male | Intact | No |  |
| Labrador Retriever | 6.63 | Female | Intact | No |  |
| Mixed breed | 8.59 | Female | Neutered | No |  |
| German Shepherd | 2.54 | Male | Neutered | No |  |
| Mixed breed | 4.11 | Female | Neutered | No |  |
| Mixed breed | 8.99 | Male | Neutered | No |  |
| German Shepherd | unknown | Male | Intact | No |  |
| Small Münsterländer | 9.92 | Male | Intact | No |  |
| Flat-coated Retriever | 10.94 | Male | Intact | No |  |
| Mini Australian Shepherd | 0.66 | Female | Intact | No |  |
| French Bulldog | 0.43 | Female | Intact | No |  |
| Swiss Mountain Dog | 9.75 | Male | Neutered | No |  |
| Mixed breed | 3.27 | Male | Neutered | No |  |
| Mixed breed | 9.42 | Male | Neutered | No |  |
| Yorkshire Terrier | 1.11 | Male | Neutered | No |  |
| Mixed breed | 6.69 | Male | Neutered | No |  |
| Golden Retriever | 0.74 | Female | Intact | No |  |
| Mixed breed | 1.9 | Female | Neutered | No |  |
| Mixed breed | 6.67 | Male | Neutered | No |  |
| German Pinscher | 8.45 | Female | Neutered | No |  |
| Mixed breed | 10.64 | Male | Intact | No |  |
| Mixed breed | 3.58 | Female | Neutered | No |  |
| French Bulldog | 0.43 | Female | Intact | No |  |
| Golden Retriever | 3.61 | Male | Chemical castration | No |  |
| Mixed breed | unknown | Female | Neutered | No |  |
| Mixed breed | 12.6 | Female | Neutered | No |  |
| Magyar Vizsla | 9 | Female | Neutered | No |  |
| Border Collie | 4.91 | Male | Intact | No |  |
| Giant Schnauzer | 9.24 | Female | Neutered | No |  |
| Mixed breed | 0.87 | Female | Neutered | No |  |

**
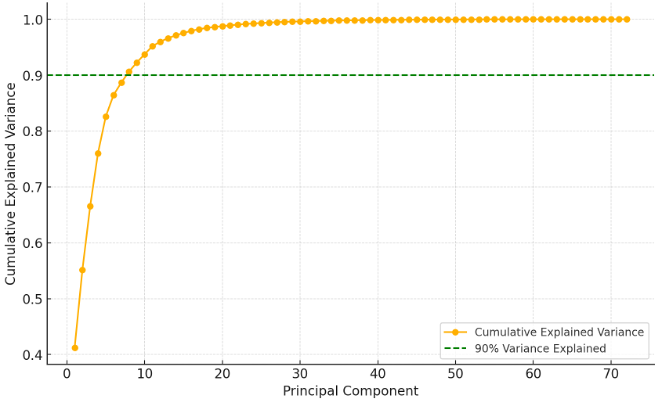
**

**Supplementary Figure 1.** Cumulative variance explained by the principal components (cutoff point: 90% of variance).

**Supplementary Table 2.** Results of ANOVA testing for differences of the first eight principal component scores (accounting for 90% variance) between the firework condition and the control condition. Significant results are bolded.

| **PC** | **F** | **Num DF** | **p** | **P corrected** |
| --- | --- | --- | --- | --- |
| PC1 | 1.603 | 1 | 0.234 | 0.719 |
| PC2 | 0.251 | 1 | 0.627 | 0.836 |
| PC3 | 0.002 | 1 | 0.960 | 0.960 |
| PC4 | 0.923 | 1 | 0.359 | 0.719 |
| **PC5** | **13.574** | **1** | **0.004** | **0.034** |
| PC6 | 0.104 | 1 | 0.753 | 0.861 |
| PC7 | 1.199 | 1 | 0.299 | 0.719 |
| PC8 | 0.535 | 1 | 0.481 | 0.770 |
